# 

**Published:** 1904-03-19

**Authors:** 


### DEATH.

Meadus.—On February 24 at The Limes, Loughton, Essex, Mary Elizabeth Meadus. (2946)

### NOTICE TO CORRESPONDENTS.

All MSS., Letters, Books for Review, and other matters intended for the Editor should be addressed The Editor, "The Hospital." The Hospital Buildings, 28 & 29 Southampton Street, Strand, W.C.

The Editor cannot undertake to return rejected MS., even when accompanied by stamped directed envelope.

### Notice to Advertisers and Subscribers.

All Advertisements, Orders for Copies of The Hospital, and Business Communications should be addressed to **THE MANAGER (Not to the Editor),** The Hospital, 28 & 29 Southampton Street, Strand, London, W.C.

The Cost of Subscription, post free, is as follows:—Per week, 1½d.; per quarter, 3s. 9d.; per half-year, 7s. 6d.; per annum, 15s. Foreign and Colonial:—Per annum, 19s. 6d., payable, in all cases, in advance.

**NOTICE.—Vol. XXXIV. of "The Hospital" (April 1903 to September 1903), in handsome cloth binding, is now on sale at the office of this Journal, price 10s. 6d. Cases for binding the Numbers contained in this Volume, 2s. Index to Vol. XXXIV., 2½d., post free.**

**The Monthly part for March is now ready. Price 1s., by post, 1s. 3d.**

As a result of the recent inquiry into the ventilation of the House of Commons it has been decided to thoroughly overhaul the arrangements and to instal new ventilating plant. Messrs. Matthews and Yates, Limited, have been entrusted with the order for this plant, and it is hoped to have it installed by the commencement of another session.

### THE STUDENT OF MEDICINE.

### APPOINTMENTS.

**John Adam**, M.B., C.M.Glasg., Visiting Physician to the Cluny Hill Hydropathic, Forres. **W. Andrew**, M.B., C.M.Edin., District Medical Officer of the Launceston Union. **E. C. Austin**, F.R.C.S.Edin., Certifying Factory Surgeon for the Broughton Astley District, Leicestershire. **Frank Barnes**, M.B., B.S., F.R.C.S., Surgical Casualty Officer to the General Hospital, Birmingham. **F. V. Bice**, M.R.C.S., L.R.C.P.Lond., District Medical Officer of the St. Austell Union. **A. Bowe**, L.R.C.P.Edin., M.R.C.S.Eng., District Medical Officer of the St. Neots Union. **E. Dykes Bower**, F.R.C.S.Edin., Medical Referee under the Workmen's Compensation Acts for the Newnham District of County Court Circuit No. 53. **H. L. Cummings**, L.R.C.P., M.R.C.S., Health Officer of Scottsdale, Tasmania. **E. E. Elliot**, M.R.C S., L.R.C.P.Lond., Medical Officer of the Dover Union Workhouse. **Francis W. E. Hare**, M.D., M.R.C.S.Eng., Visiting Medical Officer to the Diamantina Hospital for Chronic Diseases, South Brisbane. **T. J. P. Hartigan**, F.R.C.S.Eng., Assistant Surgeon to the Hospital for Diseases of the Skin, Blackfriars. **J. E. Langley**, M.R.C.S., L.R.C.P., Clinical Assistant to the Chelsea Hospital for Women. **Wm Mackay**, M.B., Ch.B.Edin., Medical Officer to the Infectious Diseases Hospital at Job's Hill, near Crook. **G. R. R. Paine**, M.R.C.S.Eng., L.S.A., District Medical Officer of the St. Thomas Union. **John Penny**, D.Sc. (Public Health), M.B., F.R.S.E., Medical Officer to the Workhouse and Medical Officer and Public Vaccinator for No. 2 District of the Cockermouth Union. **J. C. Pounden**, M.D., B.Ch.Dub., Certifying Factory Surgeon for the Alfreton District, Derbyshire, and Medical Officer for the Codner Park District of the Basford Union. **G. A. Pringle**, M.D., B.Ch.Dub., Certifying Factory Surgeon for the Aughnacloy District, Tyrone. **P. Sheehan**, L.R.C.P.&S.Edin., L.F.P.S.Glasg., District Medical Officer of the Carlisle Union. **E. G. Stocker**, L.R.C.P.Lond., M.R C.S., Certifying Factory Surgeon for the Clevedon District, Somerset. **E. W. Woodbridge**, M.B.Lond., M.R.C.S., District Medical Officer of the Barnstaple Union. **W. L. Wyatt**, M.R.C.S., L.R.C.P.Lond., District Medical Officer of the Skirlaugh Union.

The latest Cunard steamers, the *Pannonia* and *Slavonia*, have been provided with Alliott and Paton's patent high-pressure steam disinfectors, manufactured by Messrs. Manlove, Alliott and Company, Limited, engineers, Nottingham.

*JUST PUBLISHED.*

**The Sanatorium Treatment of Consumption.**

By T. N. KELYNACK, M.D., M.R.C.P.

Crown 8vo., limp cloth covers, **6d.** net, **7d.** post free.

*Of all Booksellers, or of*
THE SCIENTIFIC PRESS, Ltd., 28 & 29 Southampton Street, Strand, London, W.C.

IN THE PRESS. READY SHORTLY.

### A COMPLETE HANDBOOK OF MIDWIFERY.

By J. K. WATSON, M.D., M.B., C.M.

Author of "A Handbook for Nurses," "Examination of the Urine," &c., &c.

This handbook has been designed to meet the requirements of the Central Midwives Board, and is an up-to-date text-book of **all** that the midwife requires to know of the subject treated. The work is produced with the object of rendering it unnecessary for midwives, during their preparation for examination, to consult the larger and more expensive works on the subject written specially for medical men.

**The illustrations are very complete, numbering some 130 separate diagrams, most of which have been specially prepared for this book.**

Crown 8vo., about 350 pp., bound in cloth boards, **6/-** net, **6/4** post free.

| | | |
|---|---|---|
| 5/- net, 5/4 Post Free. | **A HANDBOOK FOR NURSES.** By J. K. Watson, M.D., M.B., C.M. New and Revised Edition. Crown 8vo. profusely illustrated, cloth gilt - - - - - - - | 5/- net, 5/4 Post Free. |
| 3/6 net, 3/10 Post Free. | **SURGICAL WARD-WORK AND NURSING.** By Alexander Miles, M.D.Edin., C.M., F.R.C.S.E. Second Edition, Enlarged and entirely Revised. Demy 8vo. copiously illustrated, cloth gilt - - - - - | 3/6 net, 3/10 Post Free. |
| 2/- Post Free. | **PRACTICAL GUIDE TO SURGICAL BANDAGING AND DRESSINGS.** By Wm. Johnson Smith, F.R.C.S. Demy 16mo. profusely illustrated (suitable for Apron pocket), cloth - - - - - - - - - | 2/- Post Free. |
| 1/- net, 1/1 Post Free. | **THE EXAMINATION OF THE URINE.** By J. K. Watson, M.D., M.B., C.M., Author of "A Handbook for Nurses." Demy 16mo. cloth boards - - - - - - - | 1/- net, 1/1 Post Free. |

*Of all Booksellers, or of*

THE SCIENTIFIC PRESS, LTD., 28 & 29 Southampton Street, Strand, London, W.C.

*Complete Catalogue of Nursing Handbooks post free on application.*

